# Relationships between psychological flexibility and internet gaming disorder among adolescents: Mediation effects of depression and maladaptive cognitions

**DOI:** 10.1371/journal.pone.0281269

**Published:** 2023-02-03

**Authors:** Xue Yang, Titus Oloruntoba Ebo, Keiman Wong, Xin Wang

**Affiliations:** 1 Center for Health Behaviours Research, JC School of Public Health and Primary Care, Faculty of Medicine, The Chinese University of Hong Kong, Hong Kong, China; 2 Department of Medicine, Faculty of Clinical Sciences, College of Medicine, University of Ibadan, Ibadan, Oyo State, Nigeria; The Hong Kong Polytechnic University, HONG KONG

## Abstract

Psychological flexibility may reduce addictive behaviours by adaptive and flexible emotional and cognitive processes. This study tested a mediation model of internet gaming disorder (IGD) in which psychological flexibility would reduce depression and maladaptive cognitions related to internet gaming and in turn lower the risk of IGD. A cross-sectional study surveyed 2102 secondary 1–4 students from seven schools during March to November 2021 in Hong Kong, China. The results showed that 12.7% and 52.2% of the students were classified as having probable IGD and depression, respectively. The proposed mediation model fitted the data well: χ^2^/df = 8.00, CFI = .99, NNFI = .99, RMSEA = .01. Psychological inflexibility was directly and positively associated with IGD (B = .01, β = .07, p = .003) and indirectly and positively associated with IGD via depressive symptoms (B = .01, β = .07, p = .001, PM = 23.7%) and maladaptive cognitions (B = .03, β = .15, p = .001, PM = 50.8%). Multi-group analyses showed that gender significantly moderated the associations between psychological inflexibility and maladaptive cognitions (Δχ^2^/Δdf = 8.69/1, p < .05), between maladaptive cognitions and IGD (Δχ^2^/Δdf = 4.33/1, p < .05), and between psychological inflexibility and IGD (Δχ^2^/Δdf = 5.46/1, p < .05). Depression and maladaptive cognitions may be significant mediators that could explain the relationship between psychological flexibility and IGD. Also, gender difference may exist. Based on the findings, intervention strategies for IGD reduction are discussed.

## Introduction

Many kinds of research have focused on internet gaming disorder and its prevalence as a result of the remarkable attractiveness of Internet games among children and teenagers around the world. An interactive game played over the Internet or through local area networks is referred to as an Internet game. The most notable examples are massively multiplayer online role-playing games, first-person shooter games, and real-time strategy games. The American Psychiatric Association has however characterized Internet Gaming Disorder (IGD) as a clinical condition that requires more research in the fifth edition, Diagnostic and Statistical Manual of Mental Disorders (DSM-5) [1, 2]. Preoccupation with gaming, development of tolerance, withdrawal symptoms, unsuccessful attempts to stop, escaping from negative moods, and compromising crucial relationships or chances due to gaming are among symptoms that people with IGD encounter. In 2022, WHO’s new International Classification of Diseases (ICD-11) comes into effect, which added “Gaming disorder, predominantly online” as a new mental health condition [3]. There is extensive evidence showing that IGD is associated with a wide range of negative outcomes among adolescents, such as physical activity, sleep, quality of life, and academic performance [4]. Due to its high prevalence and consequences especially in adolescents, IGD has emerged as a severe public health problem over the world [5–10]. For example, a study conducted in China classified 21.5% of Chinese teenage respondents as being addicted to games [11]. Another study also found a high prevalence of 15.5% in Chinese middle school students [12]. A Hong Kong study reported that 15.7% of Chinese adolescents were classified as being addicted to gaming [10]. Problematic gaming behaviours maybe intensified by the global outbreak of the coronavirus disease 2019 (COVID-19). Increasing evidence has demonstrated that Internet gaming time and the prevalence of IGD increased during school suspension and self-quarantine [13, 14]. And IGD also has been linked to COVID-19-related psychological distress among students (e.g., worry about infection and fear of COVID-19) [15]. Therefore, understanding the potential risk and protective factors of IGD is urgent and can facilitate related intervention development.

### Maladaptive cognitions and IGD

A systematic review of 36 studies reviewed a wide range of cognitions specific to internet gaming and summarized four major types of maladaptive cognitions [16, 17]. They include 1) overvaluation (the beliefs about reward value and tangibility of Internet gaming (e.g., perceptions that gaming rewards are as important as anything else); 2) maladaptive rules (justification of playing Internet games despite negative consequences); 3) gaming for self-esteem (over-reliance on Internet gaming to meet self-esteem needs); and 4) gaming for social acceptance (using Internet gaming as a way to gain social acceptance). These cognitive factors are significant motives of internet gaming behaviours. Both cross-sectional and longitudinal studies have reported that maladaptive cognitions had significantly positive associations with IGD [17–20]. Furthermore, a pilot study showed that modification of the four above mentioned domains of cognitions could reduce IGD symptoms, especially the study provides promising support for brief abstinence as a simple, practical, and cost-effective treatment technique for modifying unhelpful gaming cognitions and reducing internet gaming problems [21].

### Depression and IGD

One of the most prevalent mental health issues is depression which is also an emotional risk factor of IGD [22]. Globally, about 10% of people will experience depression at some time in their life [22]. The relationships between depression and IGD, technology addictions, and addictive behaviour have been established again and over again [23–31]. Addictive behaviours are frequently understood as external attempts to self-regulate and decrease undesirable feelings and emotional distress, which may work for a while, but it can eventually become excessive [32, 33]. Furthermore, research suggests that online addictive behaviours which include IGD serve numerous potential emotion regulation functions, such as reinforcing sensations of control, ensuring online social acknowledgment, and compensating for real-life disadvantages [24]. However, IGD activities may be desired since they are often thought to be less dangerous than other addictions [24]. Despite these theoretical constructs, there has been little research looking into the links between depression and IGD in adolescents [28, 34]. It has been stressed that depression-related developmental trajectories in emerging adulthood, as well as their interactions with IGD risk behaviours, must be considered [28].

### Psychological flexibility as a protective factor

Psychological flexibility is defined as a collection of mechanisms that enable people to cope with stress and participate in adaptive behaviours that support values-driven activity [35]. People with psychological flexibility are thought to be able to develop skills in recognizing and adapting to various contextual demands, shift their mindset or behaviours during individual and social experiences, maintain balance across important life domains, and learn to be open to, aware of, and committed to behaviours that are congruent with their values [36]. Such people also maintain a mindful awareness of their internal experiences (e.g., negative feelings, anxiety and self-doubt) if those experiences do not thwart their goal-related actions in times of stress or self-doubt. By adopting a mindful approach, psychologically flexible people are less needlessly focused on avoiding, suppressing or otherwise controlling unwanted or difficult internal experiences. This, in itself, facilitates better mental health [37, 38]. Psychological flexibility, according to many experts, is the "super talent" of resilience and mental wellness. It has been linked to increased quality of life and wellbeing, as well as being a major predictor of depression and behavioural effectiveness [39, 40]. Acceptance and Commitment Therapy (ACT) is a psychological intervention based on this theoretical framework that aims to increase psychological flexibility through six interconnected processes (acceptance, defusion, being present, self-as-context, determining a direction for behaviour change, and using techniques to facilitate change) [37]. In comparison to control conditions, such as waitlist, psychological placebo, and treatment as usual, meta-analyses have found moderate to substantial results in favour of ACT treatments in reducing depressive symptoms [41, 42]. In turn, as we mentioned above, the reduced depressive symptoms may lower the risk of IGD.

Psychological flexibility may also be a protective factor of maladaptive cognitions, thus preventing the risk of IGD. However, we did not identify any studies that examined the role of psychological flexibility in maladaptive cognitions or IGD. Maladaptive and inflexible rules about gaming were proposed to be most relevant to three criteria of IGD, the criteria being (1) the need to spend increasing amounts of time engaged in internet games, (2) unsuccessful attempts to control the participation in Internet games, and (3) continued excessive use of Internet games despite knowledge of psychological problem [2]. Within behavioural addiction theories, these three criteria are frequently symptomatic of impaired decision-making abilities [43]. Internet gaming may contain a considerable decision-making component. Excessive gaming can be sustained by sticking to many rules in the video game that allow the gamer to achieve desired goals or rationalize past decisions. This decision-making is governed by rules that lack the psychological flexibility necessary to prevent intrapersonal and interpersonal conflict. Psychologically flexible people respond to thought as a thought—an automatic verbal product of our mind—rather than as reality, or to a sensation as a sensation rather than a “negative” feeling [37, 38]. It entails viewing inner experiences for what they are, not what they say they are. Contact with the present moment means noticing events as they occur without evaluating them. It entails flexibly attending to the here and now without ruminating on the past or worrying about the future. Psychological flexibility also stresses self as context which is a process of perspective-taking, wherein the individual sees the self as a vantage point from which thoughts and feelings are observed, rather than the thoughts and feelings themselves. Thus, people with high psychological flexibility may be more likely to take a flexible and mindful manner to view themselves and internet gaming, which facilitate well-being and healthy behaviours. However, our literature search did not identify any studies that examined the inter-relationships between psychological flexibility, depression, maladaptive cognitions, and IGD.

In addition, consistent evidence shows that males are more likely to report higher a level of IGD compared to their female counterparts [20]. Moreover, the gender difference in depression and maladaptive cognitions has also been found in previous studies. It was reported that females were more vulnerable to depression than males [44], while males had higher levels of maladaptive cognitions than women [20]. However, few studies have examined whether the associations among psychological flexibility, depression, maladaptive cognitions, and IGD vary across gender. We only identified several recent studies on the gender difference in the association between depression and IGD with mixed results. For example, a study indicated that the positive association between depression and IGD was stronger in boys than girls [45]. Nevertheless, other studies found no gender differences in such an association [46, 47].

Therefore, in the present study, we proposed a mediation model of IGD in which psychological flexibility would reduce negative emotions (depression) and cognitions (maladaptive cognitions related to internet gaming) and in turn lower the risk of IGD. Specifically, it is hypothesized that 1) psychological flexibility would be associated with lower depression, maladaptive cognitions, and IGD symptoms; 2) psychological flexibility would be associated with lower IGD symptoms via reduced depression, and 3) psychological flexibility would be associated with lower IGD symptoms via reduced maladaptive cognitions. Furthermore, gender differences have been found in associations between regulation process and internalizing problems [48], it is worthwhile to investigate whether gender moderates these associations. Clarifying these factors and relationships is important because it can help to develop an effective treatment for IGD and understand the potential mechanisms of how increasing intervention related to psychological flexibility (e.g., ACT) may potentially treat and prevent IGD.

## Method

### Participants and data collection

Inclusion criteria include: (1) Secondary 1–4 students (grade 7–10); (2) Students’ and parental consent; (3) Chinese speaking. Exclusion criteria included: (1) Secondary 5–6 students due to their study pressure and preparation for public examinations. We adopted a convenient sample with seven secondary schools in Hong Kong being invited to join the study during March to November, 2021. The significance and logistics were explained in a leaflet. Parents received an information sheet; those objecting to their child’s participation could return a form to the teachers. Both the students and parents were informed about voluntary participation, and that refusal had no impact of any kind on the students. Students’ written informed consent was sought. The survey was conducted in classrooms by research staff in the absence of teachers. The questionnaire took 15 minutes to complete. Students who reported the presence of mental distress would be referred to school social workers. The participants, teachers, and parents received no material incentive. In total, 2213 were invited and 2102 completed the survey (response rate = 95%). The study procedures were carried out in accordance with the Declaration of Helsinki. Ethics approval was obtained from the Survey and Behavioural Ethics Committee of the Chinese University of Hong Kong.

### Measures

Psychological flexibility was measured by the Acceptance and Action Questionnaire-II (AAQ-II) [49]. The sample item includes, “My painful experiences and memories make it difficult for me to live a life that I would value”. The seven items are rated on a 7-point scale ranging from 1 (never true) to 7 (always true). Higher sum scores suggest greater psychological inflexibility. Previous studies examining the AAQ-II’s psychometric properties have found broad support for a one-factor model. AAQ-II has adequate reliability and validity Chinese college students [50]. The reliability was good in the current sample (Cronbach’s alpha = .88).

Maladaptive cognitions were assessed by the 24-item Internet Gaming Cognition Scale [17]. It assesses the four key previously mentioned types of maladaptive beliefs of IGD and was developed by the same researcher who developed the framework of the maladaptive beliefs on IGD [17]. Sample items include “I tend to feel better after playing Internet games” and “When I make mistakes, lose progress, or fail in an Internet game, I must reload and try again”. The items are measured by using 3-point Likert scales (0 = do not agree to 2 = strongly agree); higher scores indicate higher levels of maladaptive cognitions. The Chinese version has been validated in Chinese adolescents and showed satisfactory psychometric properties [51]. The Cronbach’s alpha of the scale was .70.

Depressive symptoms were measured by the Patient Health Questionnaire-2 (PHQ-2) [52]. The PHQ-2 inquiries about the frequency of depressed mood and anhedonia over the past two weeks. The two items are “Little interest or pleasure in doing things” and “Feeling down, depressed or hopeless”. Total scores range from 0 to 6. The cut-off point of probable depression is ≥3. The scale has been validated among Chinese students [53]. The Cronbach’s alpha of the scale was .89.

IGD symptoms were assessed by the 9-item IGD checklist is a short, user-friendly, self-report measure for assessing the DSM-5 classification of IGD [2]. Symptoms to be assessed include preoccupation, tolerance, withdrawal, unsuccessful attempts to limit gaming, deception or lies about gaming, loss of interest in other activities, use despite knowledge of harm, use for escape or relief of negative mood, and harm in the past 12 months. Response options are no (0) and yes (1). Participants who endorsed five or more items were classified as having probable IGD. Our Chinese version (the DSM-5 IGD Symptoms Checklist for Adolescents [DISCA]) was adapted and validated in Chinese adolescents [8, 54], and had high internal consistency (Cronbach’s alpha = .75).

Information of background status, including grade, gender, age, school, residency, living arrangement, perceived parental/peers’ Internet gaming behaviours, and parental education levels were self-reported by the students.

### Data analyses

Descriptive statistics were presented. T-test (for continuous variables) and chi-square test (for categorical variables) were used to compare the levels of variables between males and females. Path analyses with full information maximum likelihood estimation were conducted to test the hypothesized mediation model. Missing values were dealt with regression imputation. Several fit indices were used to indicate good model fit: (1) comparative fit index (CFI) ≥.90, (2) non-normed fit index (NNFI) ≥.90; and (3) root mean square error of approximation (RMSEA) ≤.08 [55, 56]. The direct and indirect effects and the corresponding 95% confidence interval (CI) were estimated using bootstrapping, which is a non-parametric resampling procedure that involves 5000 repeated sampling from the data set. The effect size (i.e., the proportion of mediation [PM]) was reported.

To examine the significance of each structural path across sex groups, multi-group path analyses were conducted. A series of models with each path being constrained each time were compared to the unconstrained model with all paths freely estimated using chi-square difference tests. *P* values < .05 in the chi-square difference test (Δχ^*2*^/Δdf) would denote a significant sex difference for the tested path. The SPSS 23.0 Statistics for Windows (IBM Corp. Released 2015, Armonk, NY: IBM Corp) and AMOS were used for statistical analyses.

## Result

### Description

The mean age of the participants was 13.9 years (standard deviation [SD] = 1.2) and 53.5% were male students. Most participants (80.4%) lived with both parents; 20.4% of the participants’ mothers and 23.8% of their fathers had obtained the highest educational level of college or above (Table 1). 12.7% and 52.2% were classified as having probable IGD and depression, respectively.

**Table 1:** Background variables of the participants (N = 2102).

Background characteristics	n (%) / Mean (SD)
Age	13.9 (1.2)
Sex	
Male	1125 (53.5)
Female	958 (45.6)
Missing	19 (.9)
Place of birth	
Hong Kong	1905 (90.6)
Not in Hong Kong	176 (8.4)
Missing	21 (1.0)
Living arrangement	
Live with both parents	1689 (80.4)
Live with mother only	227 (10.8)
Live with father only	82 (3.9)
Live with neither of the parents	85 (4.0)
Missing	19 (.9)
Family monthly income (HKD)	
None or < $5,001	57 (2.7)
$5,001 - $10,000	32 (1.5)
$10,001 - $20,000	158 (7.5)
$20,001 - $30,000	183 (8.7)
$30,001 - $50,000	183 (8.7)
> $50,000	159 (7.6)
Not sure	1312 (62.4)
Missing	18 (.9)
Father’s education level	
Primary or below	99 (4.7)
Secondary	884 (42.1)
College or above	501 (23.8)
Not sure	602 (28.6)
Missing	16 (.8)
Mother’s education level	
Primary or below	118 (5.6)
Secondary	949 (45.1)
College or above	430 (20.4)
Not sure	586 (27.9)
Missing	19 (.9)

### Correlations

Bivariate correlation analyses showed that all the independent variables, mediators, and dependent variables were significantly correlated with each other (range for the absolute value of r: .18-.63, all *p* < .01) (Table 2).

**Table 2:** Mean, standardized deviation (SD), correlation coefficients of the psychological variables.

	Mean	SD	1	2	3
1 Psychological inflexibility	24.53	10.13			
2 Depressive symptoms	2.76	1.64	.63**		
3 Maladaptive beliefs	12.74	8.23	.26**	.18**	
4 IGD symptoms	2.39	1.91	.30**	.26**	.62**

Note

**p < .01.

### Path analyses

**Fig 1:**
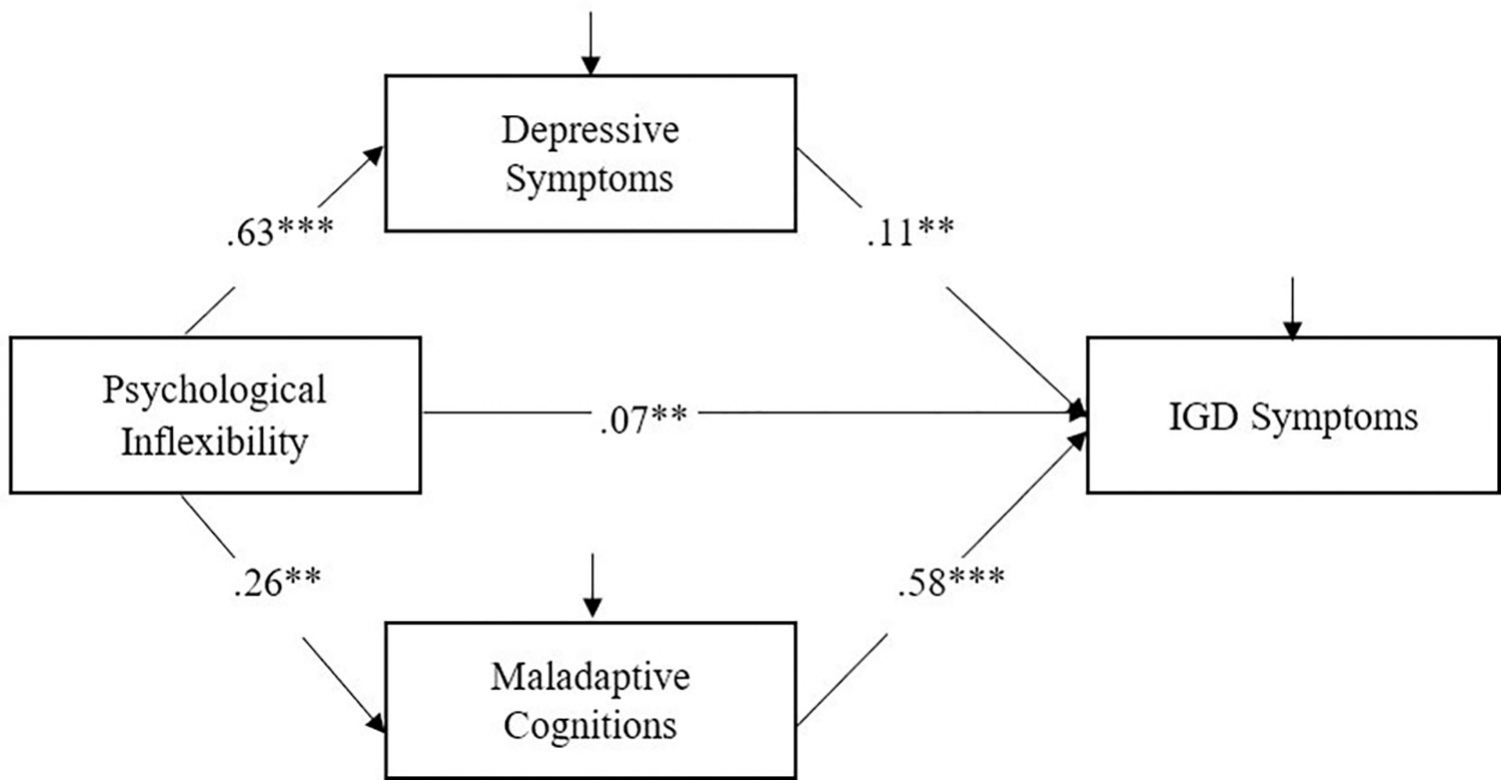
The proposed mediation model of IGD with standardized regression coefficients. Note: *p < .05, **p < .01, ***p < .001.

The proposed mediation model fitted the data well: χ^2^/df = 8.00, CFI = .99, NNFI = .99, RMSEA = .01. Psychological inflexibility was directly and positively associated with IGD (B = .01, *β* = .07, *p =* .003, 95%CI = .03 to .12). Also, psychological inflexibility was indirectly and positively associated with IGD (B = .04, *β* = .22, *p =* .004, 95%CI = .19 to .27). The specific mediation effect of depressive symptoms (B = .01, *β* = .07, *p* = .001, PM = 23.7%) and maladaptive cognitions (B = .03, *β* = .15, *p* = .001, PM = 50.8%) was statistically significant, respectively (Fig 1).

### Multi-group path analyses by gender

Multi-group path analyses showed good model fit: χ^2^/df = 3.65, CFI = .99, NNFI = .98, RMSEA = .04. Gender significantly moderated the associations between psychological inflexibility and maladaptive cognitions (Δχ^2^/Δdf = 8.69/1, *p* < .05), between maladaptive cognitions and IGD (Δχ^2^/Δdf = 4.33/1, *p* < .05), and between psychological inflexibility and IGD (Δχ^2^/Δdf = 5.46/1, *p* < .05). To be specific, the positive association between psychological inflexibility and maladaptive cognitions was stronger among males (B = .28, *β* = .32, *p* < .001) than that among females (B = .17, *β* = .25, *p* < .001). The positive association between maladaptive cognitions and IGD was weaker among males (B = .12, *β* = .51, *p* < .001) than that among females (B = .14, *β* = .63, *p* < .001). The association between psychological inflexibility and IGD was only significant among males (B = .03, *β* = .12, *p* < .001) but not among females (B = .004, *β* = .03, *p* = .45).

## Discussion

This study is the first to propose and test a model to explain whether and how psychological flexibility may reduce IGD risk through reduced depressive symptoms and maladaptive cognitions. The proposed mediation model was supported by data, and gender differences in the model paths were identified. Although psychological flexibility has been demonstrated to predict a range of psychological problems and addictive behaviours, including substance use disorders, this study is the first attempt to extend the psychological flexibility model to understand IGD.

Depression has been well-documented as a consequence of a lack of psychological flexibility [39, 40] and a risk factor of IGD [23–29]. This mediation effect of depression highlights the emotional mechanism in understanding the relationship between psychological flexibility and IGD risk. Indeed, psychological flexibility involves people deliberately observing their internal experiences on a moment-to-moment basis, in an open, non-elaborative, non-controlling, and non-judgmental manner [37, 38]; it is also associated with emotion intelligence and emotion regulation [57]. Thus, psychologically flexible people may be less likely to experience negative and fearful emotions and feelings or adopt avoidance coping strategies, such as using Internet gaming to escape from real-world stress. Other emotions (e.g., anxiety, joy) should be tested in future work to better understand the emotional mechanisms between psychological flexibility and IGD.

On the other hand, maladaptive cognitions may be a cognitive mechanism that explains the relationship between psychological flexibility and IGD risk. Psychological flexibility may improve one’s awareness of inner experience and cognitive process [37, 38]; thus, psychological flexibility may reduce cognitive bias related to Internet gaming (e.g., over-reliance on Internet gaming to meet self-esteem needs), which in turn may reduce IGD risk. Interestingly, we found that these model paths may be gender-specific; the effect of psychological flexibility on maladaptive cognitions was stronger among males, while the effect of maladaptive cognitions on IGD was stronger among females. It may suggest that males with psychological inflexibility were more likely to develop maladaptive thoughts related to Internet gaming, while females who possessed these thoughts might be more susceptible to developing IGD than their male counterparts possessing the same thoughts. In other words, enhancing psychological flexibility may be more effective to reduce males’ cognitive bias about internet gaming; however, directly targeting such cognitive bias among females may be more effective in reducing their IGD symptoms.

The significant direct effect of psychological flexibility on IGD among males but not females may suggest that there exist other mediators/mechanisms between this association and they may be gender-specific. For example, males are more likely to have risk-taking behaviours and impulsivity [58, 59] which may be potential mediators; that is, psychological flexibility may be particularly important in reducing males’ risk-taking tendency and impulsivity, thus reducing their IGD risk. Other potential mediators, such as maladaptive coping styles and experiential avoidance, also may explain the relationship between psychological flexibility and IGD as psychological flexibility may reduce such negative behavioural tendency. Gender differences and potential mediators in these relationships should be further explored in future work. Qualitative interviews may be helpful to explore other potential moderators and mediators between psychological flexibility and IGD and other triggers and protective factors of IGD. Such information will help researchers to develop a comprehensive model of IGD.

Mediation models provide one method for obtaining more refined support for underlying theoretical models and principles by examining whether changes in outcome are functionally related to changes in theoretical processes [60]. It helps to determine which components of which treatments work best for whom. Our findings support the use of ACT by elucidating a potential relevance of psychological flexibility to IGD symptoms across gender and revealing the potential underlying mechanisms. ACT is a treatment that explicitly attempts to increase psychological flexibility, which is an effective treatment option for substance use disorders (e.g., alcohol dependence) and non-substance use disorders (e.g., gambling) [61, 62]. ACT may reduce IGD by helping the clients to accept their negative emotions and cognitive bias related to internet gaming in a non-judgmental way, instead of using avoidance coping or fighting with these negative experiences. Furthermore, ACT helps the clients to identify and commit to their values and take actions towards living a meaningful life. In addition to ACT, cognitive behavioural therapy (CBT) has also demonstrated its efficacy in reducing depression, cognitive bias in general, and IGD symptoms [63]. The core CBT strategies for IGD aim to enhance clients’ self-regulation and positive coping strategies, rather than relying on internet gaming to escape from real life stress, and to reduce cognitive bias related to internet gaming. Strategies, such as self-monitoring, challenging or disputing beliefs, and problem-solving, improvement of interpersonal relationships and communication skills [16], in CBT may be particularly effective to reduce depression and cognitive bias. Both ACT and CBT have been recommended to address multiple mental problems based on a transdiagnostic approach [64, 65]. They have a potential to enhance cost-effectiveness of the treatment when reducing both IGD and depression among adolescents. The need and urgency of reducing IGD and depression have been highlighted by their high prevalence in this study. These results are similar with those found among Hong Kong adolescents in 2020 (15% and 60%) during COVID-19 [10] and those reported before COVID-19 (13% and 57%) [66, 67]. It seems that the prevalence of probable IGD and depression did not increase substantially during the pandemic. Continuous surveillance at different phases of the pandemic is warranted to monitor the changes of these problems over time and prevent the potential long-term impact of COVID-19 on adolescents’ mental and behavioural health.

In spite of the promising results, several limitations of this study should be noted. First, the approach of convenience sampling was used in this study. Thus, the generalizability of the findings may be limited. Future studies should validate the results in a representative sample, other age groups, and other cultures. Second, there may be reporting bias and social desirability bias due to the non-anonymous survey and self-reported measurements. Third, we could not make causal inferences due to the cross-sectional design. Last but not least, to shorten the questionnaire and reduce the burden of the adolescent participants, we used the short form of PHQ-9, PHQ-2, to assess depression. Future studies should validate the results by using the full version scale to measure depression.

To conclude, the present study investigated a mediation model to illustrate the relationship between psychological flexibility and IGD via emotional and cognitive processes and the moderation effect of gender. Based on these findings, ACT and CBT that can enhance psychological flexibility and reduce negative emotions and maladaptive cognitions may have the potential to reduce IGD. Longitudinal studies are warranted to verify the present findings. Qualitative interviews may be helpful to explore other potential moderators and mediators between psychological flexibility and IGD and other triggers and protective factors of IGD. Such information will help researchers to develop a comprehensive model of IGD.

## Supporting information

S1 File (SAV)
